# Whole-Genome Sequencing Exhibits Better Diagnostic Performance than Variable-Number Tandem Repeats for Identifying Mixed Infections of Mycobacterium tuberculosis

**DOI:** 10.1128/spectrum.03570-22

**Published:** 2023-04-26

**Authors:** Yiwang Chen, Qi Jiang, Qingyun Liu, Mingyu Gan, Howard E. Takiff, Qian Gao

**Affiliations:** a National Clinical Research Center for Infectious Diseases, Shenzhen Third People’s Hospital, Shenzhen, Guangdong, China; b Key Laboratory of Medical Molecular Virology (MOE/NHC/CAMS), School of Basic Medical Sciences, Shanghai Medical College, Fudan University, Shanghai, China; c School of Public Health, Public Health Research Institute of Renmin Hospital, Wuhan University, Wuhan, China; d Department of Immunology and Infectious Diseases, Harvard T. H. Chan School of Public Health, Boston, Massachusetts, USA; e Molecular Medical Center, Children’s Hospital of Fudan University, Shanghai, China; f Instituto Venezolano de Investigaciones Cientificas (IVIC), Caracas, Venezuela; American Type Culture Collection

**Keywords:** VNTR, detection sensitivity, mixed infections, retreatment tuberculosis cases, whole-genome sequencing

## Abstract

Mixed infections of Mycobacterium tuberculosis, defined as the coexistence of multiple genetically distinct strains within a single host, have been associated with unfavorable treatment outcomes. Different methods have been used to detect mixed infections, but their performances have not been carefully evaluated. To compare the sensitivity of whole-genome sequencing (WGS) and variable-number tandem repeats (VNTR) typing to detect mixed infections, we prepared 10 artificial samples composed of DNA mixtures from two strains in different proportions and retrospectively collected 1,084 clinical isolates. The limit of detection (LOD) for the presence of a minor strain was 5% for both WGS and VNTR typing. The overall clinical detection rate of mixed infections was 3.7% (40/1,084) for the two methods combined, WGS identified 37/1,084 (3.4%), and VNTR typing identified 14/1,084 (1.3%), including 11 also identified by WGS. Multivariate analysis demonstrated that retreatment patients had a 2.7 times (95% confidence interval [CI], 1.2 to 6.0) higher risk of mixed infections than new cases. Collectively, WGS is a more reliable tool to identify mixed infections than VNTR typing, and mixed infections are more common in retreated patients.

**IMPORTANCE** Mixed infections of M. tuberculosis have the potential to render treatment regimens ineffective and affect the transmission dynamics of the disease. VNTR typing, currently the most widely used method for the detection of mixed infections, detects mixed infections only by interrogating a small fraction of the M. tuberculosis genome, which necessarily limits sensitivity. With the introduction of WGS, it became possible to study the entire genome, but no quantitative comparison has yet been undertaken. Our systematic comparison of the ability of WGS and VNTR typing to detect mixed infections, using both artificial samples and clinical isolates, revealed the superior performance of WGS at a high sequencing depth (~100×) and found that mixed infections are more common in patients being retreated for tuberculosis (TB) in the populations studied. This provides valuable information for the application of WGS in the detection of mixed infections and the implications of mixed infections for tuberculosis control.

## INTRODUCTION

Mixed infections of Mycobacterium tuberculosis (M. tuberculosis), defined as the coexistence of multiple distinct strains within the same patient, have attracted increasing attention because they are a potential source of clinical and public health problems (1). Prior studies have suggested that mixed infections with strains of different resistance phenotypes can complicate drug-susceptibility testing (DST) because the resistance present in the minority strain may not be detected (2, 3). As a result, a drug regimen based on DST results that only detect the majority strain could be inappropriate and lead to treatment failure. Cohen et al. (4) used mathematical models to show that mixed infections could also compromise the expected benefits of new antituberculosis vaccines if they do not adequately cover all circulating strain types. Mixed infections can also cause M. tuberculosis transmission links to be missed if an undetected strain in a patient with a mixed infection is transmitted to another patient (5, 6).

Current approaches to identify mixed infections employ molecular genotyping methods and whole-genome sequencing (WGS). The classical molecular genotyping methods include spoligotyping (7), IS*6110* restriction fragment length polymorphism (RFLP) typing (8), and variable-number tandem repeat (VNTR) typing (9). Among these, VNTR typing is currently the most widely used method because of its efficiency and simplicity (1, 10). However, VNTR typing examines heterogeneity within only a limited set of loci, which limits its sensitivity. WGS offers superior resolution compared to molecular typing methods (11) and is increasingly used to detect mixed M. tuberculosis infections (12, 13). In this report, we compared the accuracy of WGS and VNTR for detecting mixed infections in artificial and clinical samples and identified risk factors for mixed infections. This work should provide theoretical background for further understanding mixed infections and developing effective control strategies.

## RESULTS

### The limit of detection (LOD) of WGS and VNTR typing for mixed infections.

**FIG 1:**
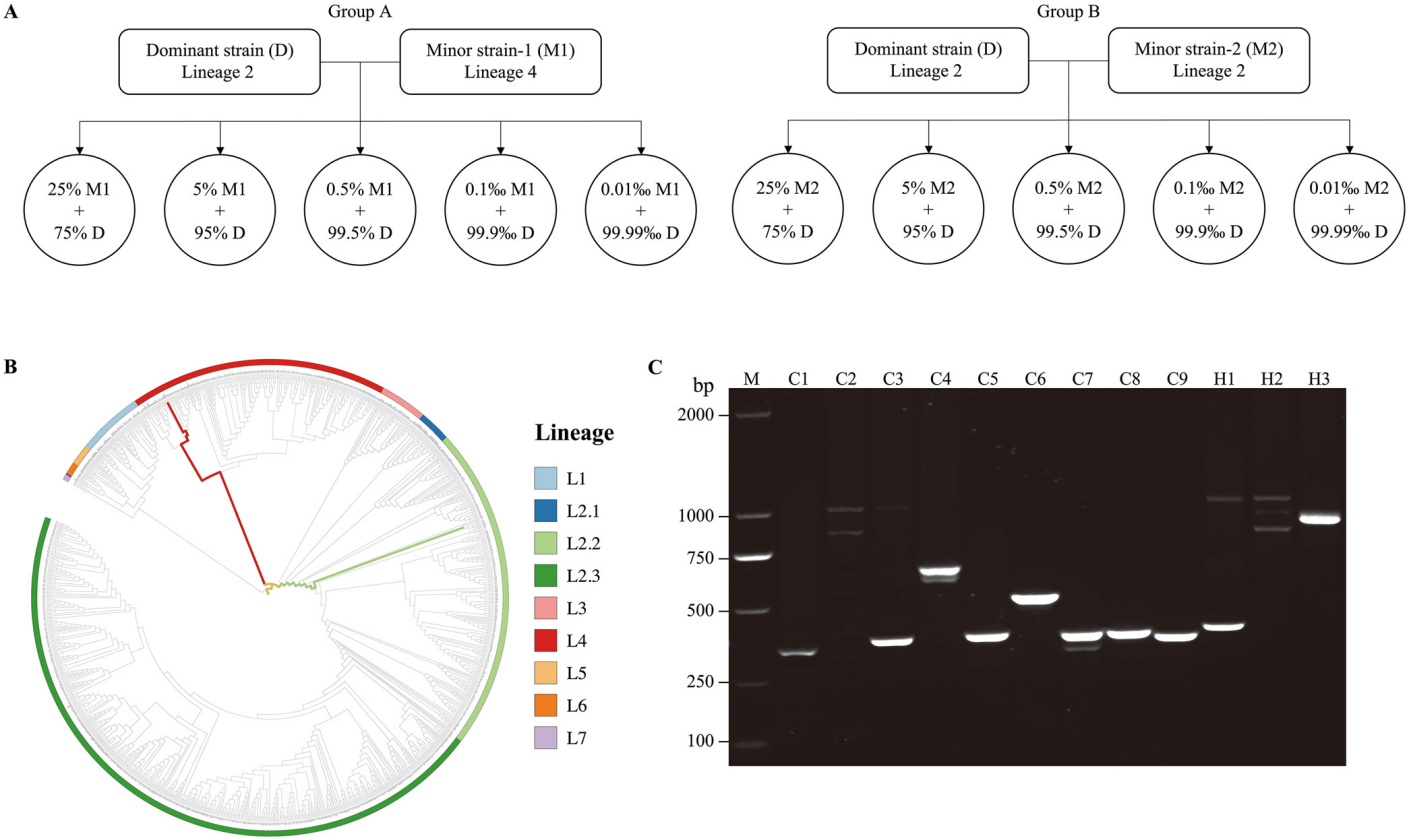
Schematic of the preparation of laboratory samples used to simulate mixed infections of M. tuberculosis and two methods for detection of mixed infections. (A) Preparation of laboratory mixed samples containing two strains belongs to distinct (group A) and same (group B) lineages. (B) Two evolutionary paths were determined by mapping SNPs called from group A mixed samples to the reference phylogenomic database. (C) PCR amplification of multiple VNTR loci from group B mixed samples generated two bands. C1 to C9 indicate the 9 conventional loci and H1 to H3 indicate the 3 hypervariable loci. M, marker.

**FIG 2:**
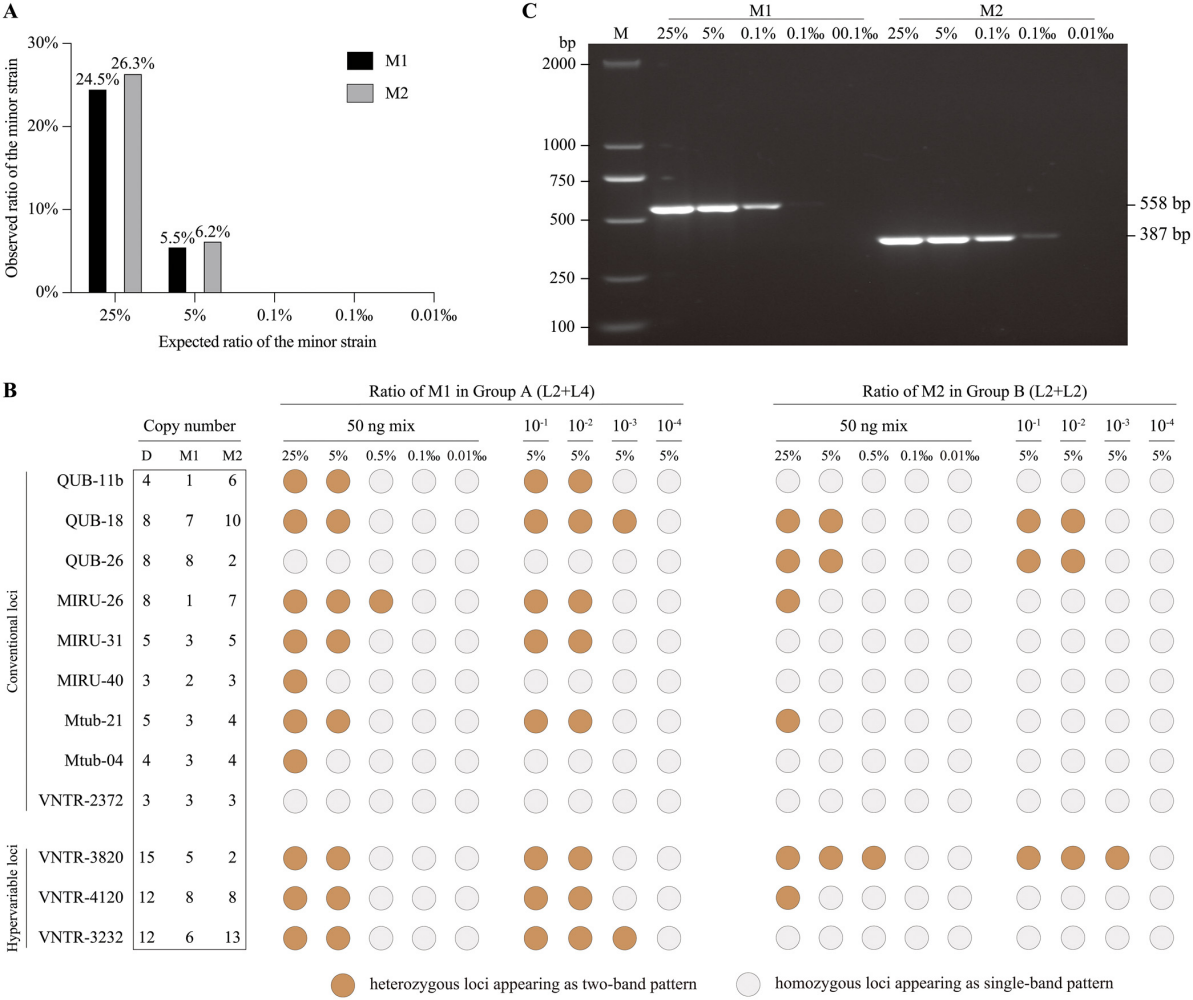
Detection of mixed infections by VNTR typing and WGS among laboratory mixed samples. (A) VNTR patterns of laboratory mixed samples and their 10-fold serial dilutions from group A and group B. (B) Detection of mixed infections from WGS data of artificial mixed samples from group A and group B. (C) PCR amplification of the VNTR loci (VNTR-3820) of the minor strain alone, based on DNA quantities corresponding to the relevant mixed samples. D, dominant strain; M1, minor strain-1; M2, minor strain-2; M, marker.

We first investigated the sensitivity of WGS and VNTR typing for detecting mixed infections in artificial samples containing DNA mixtures in various proportions from two M. tuberculosis strains belonging to the same (group A) or different (group B) phylogenetic lineages (Fig. 1A). Sequencing libraries were constructed using the minimum genomic DNA (50 ng, ~1.1 × 10^7^ genomes) recommended by the manufacturer. WGS analysis revealed that the average sequencing depth of these artificial samples was 134× (105 to 171×), with an average genomic coverage of 99.5%. The LOD for mixed infections was the presence of each strain in a proportion of at least 5% (~5.5 × 10^5^ genomes). This 5% LOD was found in mixtures from both groups A and B, and the ratio of the two different strains in the WGS data was consistent with the actual mixing ratio (Fig. 2A). VNTR typing, using the same quantity of genomic DNA as used for WGS, similarly had a LOD for mixed infection of 5% in the artificial samples from both groups A and B (Fig. 2B; see Fig. S2 in the supplemental material).

### Competition in PCR affected the LOD of VNTR typing for mixed infections.

Previous studies have shown that the minimum amount of template DNA for PCR amplification of the VNTR loci is about 440 copies of the genome(14), but we found that the minor strain was undetectable when it was present in the artificial samples at ratios less than 5%, which is calculated to represent about 5.5 × 10^5^ genome copies. To investigate the minimum amount of template DNA required for VNTR to identify mixed infections, we performed 10-fold dilutions of the artificial samples. We found that when the minor strain was present in a proportion of at least 5%, it was still detected when the 50 ng total DNA in artificial mixtures A and B was diluted 10^−3^ and 10^−2^, respectively, although the number of heterozygous loci decreased as the quantity of template DNA was reduced (Fig. 2B, Fig. S2).

PCR amplification of different DNA fragments flanked by the same primer sites can result in competition between the targets (15). We therefore speculated that competition in PCR might lead to a decreased LOD for detecting mixed infections using VNTR. To test this possibility, we determined the minimum amount of DNA required for VNTR amplification when only the minor strain was present in group A and B samples. PCR amplification of the minor strains alone was achieved with a minimum of 0.005 ng of DNA (1.1 × 10^3^ genomes), which corresponds to 0.01‰ (0.005 ng/50 ng) of the total DNA present in the mixed artificial samples (Fig. 2C).

### Identification of mixed infections in clinical M. tuberculosis isolates.

**FIG 3:**
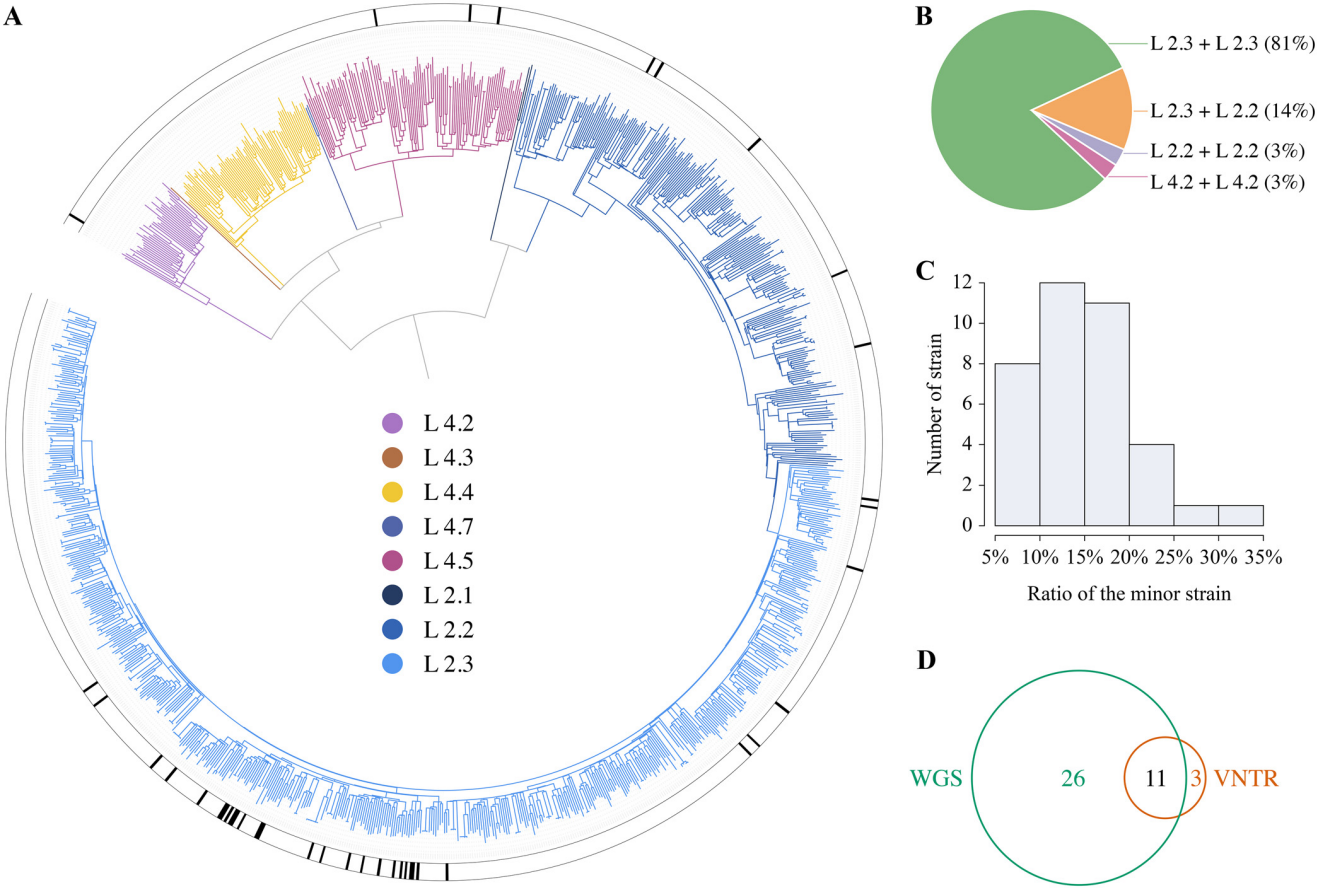
Detection of mixed infections by WGS and VNTR typing among clinical isolates. (A) The phylogenetic tree of 1,084 Mycobacterium tuberculosis strains isolated in the Songjiang District of Shanghai, China, from 2011 to 2015. The different colors on the branches indicate different phylogenetic lineages; the outside circle indicates 40 isolates with mixed infections detected by WGS and VNTR. (B) Pie chart of different combinations of sublineages from 37 isolates detected with mixed infections by WGS. (C) Ratio distribution of the minor strain from 37 isolates detected with mixed infections by WGS. (D) Venn diagram of the numbers of isolates with mixed infections identified by WGS and VNTR.

To compare the ability of WGS and VNTR typing to detect mixed infections in clinical isolates, we analyzed the isolates grown from pretreatment sputum samples of 1,084 tuberculosis (TB) patients. Phylogenetic analysis based on their WGS data (Fig. 3A, Table 1) showed that they belonged predominantly to the Beijing family (79%, 856/1,084). Molecular DST results showed that there were 130 (12%) strains resistant to at least one anti-TB drug, including 48 multidrug-resistant (MDR)-TB strains.

**TABLE 1:** Demographic, clinical, and bacteriological characteristics of patients detected with mixed infections of Mycobacterium tuberculosis

Characteristic	All infections (*n* = 1,084)		Non-mixed infection (*n* = 1,044)		Mixed infection (*n* = 40)		*P* value
	No.	%	No.	%	No.	%	
Migratory behavior							0.920
Resident	251	23.2	242	23.2	9	22.5	
Migrant	833	76.8	802	76.8	31	77.5	
Gender							0.724
Female	353	32.6	341	32.7	12	30.0	
Male	731	67.4	703	67.3	28	70.0	
Age, yrs							0.127
15–24	359	33.1	345	33.0	14	35.0	
25–44	433	39.9	423	40.5	10	25.0	
45–64	185	17.1	174	16.7	11	27.5	
≥65	107	9.9	102	9.8	5	12.5	
Occupation							0.937
Labor worker	581	53.6	561	53.7	20	50.0	
Housekeeper	174	16.1	167	16.0	7	17.5	
Farmer	89	8.2	86	8.2	3	7.5	
Other	240	22.1	230	22.0	10	25.0	
Treatment history							0.046
New	952	91.2	920	88.1	32	80.0	
Retreatment	86	8.2	78	7.5	8	20.0	
Unknown	6	0.6	6	0.6	0	0.0	
Diagnosis delay							0.841
≤2 wks	209	19.3	200	19.2	9	22.5	
2–4 wks	370	34.1	357	34.2	13	32.5	
4–8 wks	294	27.1	285	27.3	9	22.5	
>8 wks	207	19.1	198	19.0	9	22.5	
Unknown	4	0.4	4	0.4	0	0.0	
Treatment outcome							0.012
Favorable	959	88.5	927	88.8	32	80.0	
Unfavorable	43	4.0	43	4.1	0	0.0	
Unknown	82	7.6	74	7.1	8	20.0	
Cavitation							1.000
Yes	642	59.2	618	59.2	24	60.0	
No	428	39.5	412	39.5	16	40.0	
Unknown	14	1.3	14	1.3	0	0.0	
Sputum smear status							0.262
Positive	652	60.1	623	59.7	29	72.5	
Negative	417	38.5	406	38.9	11	27.5	
Unknown	15	1.4	15	1.4	0	0.0	
Drug resistance profile							0.177
Pan-susceptible	954	88.0	918	87.9	36	90.0	1.000
Multidrug resistance	48	4.4	47	4.5	1	2.5	
Other drug resistance	82	7.6	79	7.6	3	7.5	
Beijing strain							0.081
Yes	856	79.0	820	78.5	36	90.0	
No	228	21.0	224	21.5	4	10.0	

WGS identified mixed infections in 37 isolates (3.4%, 37/1,084), 30 of which (81.1%, 30/37) contained two modern Beijing strains (L2.3 and L2.3; Fig. 3B). The minor strain was present in a proportion of 25% or less in 94.6% (35/37) of the mixed infections (Fig. 3C), and no heteroresistance was detected in any of these 37 isolates. VNTR typing detected 14 isolates (1.3%, 14/1,084) with mixed infections: 1 isolate had 4 heterogeneous VNTR loci, 1 had 3 heterogeneous VNTR loci, and the remaining 12 isolates had 2 heterogeneous loci. Combining WGS and VNTR results, mixed infections were detected in a total of 40 isolates (3.7%, 40/1,084). WGS exhibited a higher detection rate than VNTR typing (3.4% versus 1.3%, *P* = 0.001), but only 78.6% (11/14) of mixed infections identified by VNTR typing were also detected by WGS (Fig. 3D).

### Analysis of risk factors for mixed infections.

To identify the risk factors associated with mixed infections, we compared the demographic, clinical, and bacteriological characteristics of cases with and without mixed infections (Table 1). The proportion of retreatment cases was much higher among the 40 patients with mixed infections than in the patients infected with a single strain (20.0% versus 8.2%, *P* = 0.046). Beijing strains were slightly, but not significantly, more frequent in patients with mixed infections than in other patients (90.0% versus 79.0%, *P* = 0.081). In the multivariate logistic analysis, adjusted by Beijing genotype, retreatment patients had 2.7 times (95% confidence interval [CI], 1.2 to 6.0) the risk of mixed infections as new cases.

## DISCUSSION

In this study, we compared the capacity of whole-genome sequencing and VNTR typing to detect mixed infections. Based on artificial samples, we found that both WGS and VNTR typing could detect mixed infections when the genomic DNA from the minority strain was present in a proportion of at least 5%. Combining the results from both methods analyzing 1,084 clinical isolates, the total proportion of mixed infections was 3.7% (40/1,084), with WGS detecting more mixed infections than VNTR typing (37/1084, 3.4% versus 14/1,084, 1.3%). The concordance between the two methods was 97.3% (1,055/1,084). Multivariable analysis showed that retreated patients were at significantly higher risk of having a mixed infection than new cases.

The main factors affecting the sensitivity of VNTR and WGS for detecting mixed infections were the genomic detection range and the completeness of the reference evolutionary tree. WGS can provide comprehensive genetic information and therefore has a higher resolution than VNTR, which is based on limited genomic regions (1, 10), and this higher resolution implies higher sensitivity to detect genomic differences (16). Many studies of molecular epidemiology have shown that the clustering rate determined by WGS is lower than that determined by VNTR because WGS is better able to discriminate between similar but distinct strains (17, 18). However, because the identification of mixed infections by WGS depends on identifying two distinct mutational phylogenetic paths that are present in the database (12), if the divergent paths of the two strains are not included in the database, the mixed infection will not be detected (Fig. S1). The three cases of mixed infections identified by VNTR but missed by WGS illustrate that the phylogenetic-based WGS method used in this study is not perfect. The sensitivity of this method for detecting mixed infectious could perhaps be improved if the reference evolutionary tree incorporated more local strains.

Recently, many other WGS-based methods have been developed for the detection of mixed infections. MixInfect (19) is a method for mixed M. tuberculosis infection estimation that uses a Bayesian model-based clustering technique. While this method can estimate mixture proportions, it does not provide any functionality for resolving the constituent strains. QuantTB, described by Anyansi et al. (13), quantifies the multiplicity and abundance of mixed M. tuberculosis infections based on a reference database. Similar to our phylogenetic method, its performance is highly dependent on the database’s representation of the common strains in the relevant local context. Gabbassov et al. (20), developed a tool called SplitStrains that is grounded in a rigorous statistical framework. This tool can estimate the M. tuberculosis mixture proportions and partially reconstruct their sequences using an expectation-maximization (EM) algorithm. However, the EM algorithm has a very slow convergence, and it makes the convergence to the local optima only. In addition, several metagenomic tools exist to classify mixed populations of strains within a single species (21), but these methods were not designed to be able to discriminate between strains of highly clonal species such as M. tuberculosis.

Although we determined that the LOD for mixed infections using both WGS and VNTR was 5%, the limiting factors of the two methods are different. For WGS, the LOD for mixed infections depends on its sequencing depth (22). The average sequencing depth in this study was about 100×, and single nucleotide polymorphisms (SNPs) with frequencies below 5% were eliminated to exclude possible sequencing errors (23). In contrast, Gan et al. (12) used deep WGS with a depth of ~1,000× and reported that the LOD for mixed infections was 0.64%. Although this depth (1,000×) is currently not routine when performing WGS of many M. tuberculosis isolates, it could become feasible with improved sequencing technology, thereby reducing the LOD for detecting mixed infections. For PCR-based VNTR typing, despite the high sensitivity of PCR amplification for a single strain, the PCR target competition in mixed infections appears to be an obstacle to lowering the LOD.

Previous studies have reported different prevalences of mixed infections, but they employed different methods and study designs. Our finding that WGS is more sensitive for detecting mixed infections suggests that reports based only on VNTR typing may have underestimated the prevalence of mixed infections (8). In contrast, Fang et al. (24), using VNTR typing, reported mixed infections in 5.6% of clinical isolates in Shanghai, China, higher than the 3.7% we found using both WGS and VNTR. The difference may be because their definition of a mixed infection required only one heterogenous locus, while our definition required two (25). Peng et al. (26) analyzed two sputum samples per patient and detected mixed infections in 11.2% of TB cases in Heilongjiang, China, but the proportion of mixed infections decreased to 5.6% when the analysis was based on only one sample. This is consistent with recent findings that sequential sputum samples can have different representations of in-host bacterial diversity (27).

WGS, although not perfect, was superior to VNTR for identifying mixed infections. Another advantage of WGS is that the data can be easily stored, subjected to additional analyses in the future, and compared with results from different laboratories (11). This will provide a foundation for further research not only on the detection of mixed infections but also on their significance and why they occur. For example, most previous studies have focused on mixed infections caused by strains with different resistance profiles, but more research is needed on the clinical implications of mixed infections with strains having the same resistance profile (9, 28). For example, do the two strains have distinct transmission potentials that could be explained by identifying determinants in the bacterial genome (29)? Are there host or environmental factors that predispose patients to mixed infections (30, 31)? These questions may be amenable to analysis with WGS. Furthermore, we detected no mixed infections involving different lineages, which may indicate that different lineage types could vary in their susceptibility to mixed infections. The simultaneous interaction between multiple strains and the host immune system in a mixed infection scenario remains to be explored.

This study had several limitations. The inconsistencies in the performance of WGS and VNTR for detecting mixed infections in laboratory versus clinical samples may be due to insufficient strain genotype combinations in the artificial samples; e.g., there were no combinations of strains that cannot be classified by VNTR typing. Mixed infections detected by WGS should be further confirmed using additional methods, such as single-colony sequencing.

In conclusion, our systematic comparison of the ability of WGS and VNTR to detect mixed infections, using both artificial samples and clinical isolates, revealed the superior performance of WGS at a high sequencing depth (~100×) and found that mixed infections are more common in patients being retreated for TB in the populations studied.

## MATERIALS AND METHODS

### Preparation of artificial samples and selection of clinical M. tuberculosis isolates.

Genomic DNA was extracted in the biosafety level 2 plus (BSL-2+) laboratory from M. tuberculosis strains using the cetyltrimethyl-ammonium bromide-lysozyme (CTAB) method (32). Each artificial sample was composed of mixtures of DNA from two strains in different proportions (Table S1). Two groups of DNA mixtures were prepared (Fig. 1A): group A contained DNAs from two strains belonging to different lineages (L2 and L4) that were separated by a genetic distance of 1,159 single nucleotide polymorphisms (SNPs); group B contained DNAs from two strains belonging to the same lineage (L2) that had a genetic distance of 392 SNPs. DNAs from the two strains were diluted separately in double-distilled water (ddH_2_O) to a final concentration of 50 ng/μL and mixed in each of the following proportions: 25%:75%, 5%:95%, 0.5%:99.5%, 0.1‰:99.9‰, and 0.01‰:99.99‰. We also prepared 10 additional samples consisting of only the minor strain in group A (M1) and B (M2) samples, with the same concentrations of the strain DNA as in the artificial mixtures.

The Songjiang District is located in the southwest of Shanghai, China, and has an incidence of pulmonary tuberculosis (TB) of approximately 33.6/100,000. All suspected TB cases in Songjiang are referred to the local TB-designated hospitals, and all culture-positive M. tuberculosis isolates are collected and stored in the Shanghai Municipal Centre for Disease Control and Prevention (SCDC). The SCDC provided our study with all 1,084 stored isolates cultured from the sputum samples of TB patients diagnosed from 2011 to 2015 in the Songjiang District, before initiating treatment.

### Analysis of WGS data.

Paired-end sequencing libraries were constructed with 300-bp inserts, and 150-bp paired-end reads were generated on an Illumina HiSeq 2500 platform. A previously described pipeline was used for calling single nucleotide polymorphisms (SNPs) (27). Briefly, the Sickle tool was used to trim WGS data to keep reads with a Phred base quality above 20 and lengths greater than 30 nucleotides. Reads were mapped to the M. tuberculosis H37Rv reference strain (GenBank AL123456) with Bowtie 2 software (version 2.2.9), and then SAMtools (version 1.3.1) was used for SNP-calling with a mapping quality greater than 30. Varscan (version 2.3.9) was used to identified fixed (frequency, ≥ 95%) and unfixed (<95%) SNPs with at least 5 supporting reads and the strand bias filter option on. SNPs in repetitive regions of the genome (e.g., the proline-glutamic acid (PE)/proline-proline-glutamic acid (PPE) polymorphic guanine-cytosine-rich sequence (PE/PPE-PGRS) family genes, phage sequences, insertion, or mobile genetic elements) were excluded. The drug resistance profiles and lineages of the M. tuberculosis strains were predicted from WGS data using SAM-TB (33). Multidrug-resistant tuberculosis (MDR-TB) was defined as isolates with mutations conferring resistance to at least isoniazid and rifampicin. Samples with unfixed drug-resistant mutations (DRMs) in at least one locus were considered heteroresistant. Phylogenetic trees were constructed using the maximum-likelihood method (RAxML-NG) (34) and visualized on the Interactive Tree of Life platform (https://itol.embl.de/). Files containing sequencing reads were deposited in the National Institutes of Health Sequence Read Archive under BioProject PRJNA869190.

### Detection of mixed infections.

WGS data were used to identify mixed infections using the phylogenetic method of Gan et al. (12), which is based on detecting two evolutionary paths. The evolutionary path of an M. tuberculosis strain can be determined by mapping its SNPs onto a reference phylogeny computed from published M. tuberculosis genome sequences (Fig. 1B). The proportion of mixed infections was obtained by calculating the arithmetic mean of the mutation frequencies based on the unique mutations matched in each pathway (Fig. S1).

VNTR typing was performed using a 9+3 locus combination that has a high resolution for Beijing lineage strains (25). After extraction of bacterial genomic DNA by boiling, nine conventional loci (QUB11b, QUB18, Mtub21, Miru26, QUB26, Mtub04, Miru31, Miru40, and VNTR 2372) and three hypervariable loci (VNTR 3820, 3232, and 4120) were separately amplified by PCR using the primers listed in Table S2. The PCRs for the nine conventional loci were performed in a volume of 10 μL containing 1× *Taq* PCR master mix (CoWin Biotech Co. Ltd., Beijing, China), 0.4 μM each primer, and 1 μL DNA template. The reactions for the three hypervariable loci were performed in a volume of 20 μL containing 1× GC buffer I (TaKaRa Biotech Co. Ltd., Dalian, China), 200 μM each deoxynucleoside triphosphate (dNTP), 0.5 U of *Taq* (TaKaRa Biotech Co. Ltd.), 0.4 μM each primer, and 1 μL DNA template. The thermocycling conditions were as follows: 95°C for 5 min, followed by 30 cycles at 94°C for 30 s, 58°C (64°C for loci MIRU 40 and VNTR 4120) for 30 s, and 72°C for 30 s (1.5 min for loci VNTR 3232, VNTR 3820, and VNTR 4120), with a final extension at 72°C for 7 min. Based on the PCR results, a locus was considered heterozygous if the PCR product showed at least 2 bands on agarose gel electrophoresis (Fig. 1C). If a sample had two or more heterozygous loci, it was considered a mixed infection (9).

### Statistical analysis.

The basic demographic and clinical data for the enrolled TB patients were extracted from the routine TB surveillance system. The chi-square test was used to assess possible associations of mixed infections. Univariable and multivariate logistic regressions were used to estimate the odds ratios (ORs) and 95% confidence intervals (CIs). All statistical analyses were performed and visualized in R (version 3.6.0), with *P* values of <0.05 considered statistically significant.

## References

[1] Cohen T, van Helden PD, Wilson D, Colijn C, McLaughlin MM, Abubakar I, Warren RM. 2012. Mixed-strain Mycobacterium tuberculosis infections and the implications for tuberculosis treatment and control. Clin Microbiol Rev 25:708–719. doi:10.1128/CMR.00021-12. 23034327 PMC3485752

[2] Zetola NM, Shin SS, Tumedi KA, Moeti K, Ncube R, Nicol M, Collman RG, Klausner JD, Modongo C. 2014. Mixed Mycobacterium tuberculosis complex infections and false-negative results for rifampin resistance by GeneXpert MTB/RIF are associated with poor clinical outcomes. J Clin Microbiol 52:2422–2429. doi:10.1128/JCM.02489-13. 24789181 PMC4097703

[3] Kargarpour Kamakoli M, Sadegh HR, Farmanfarmaei G, Masoumi M, Fateh A, Javadi G, Rahimi Jamnani F, Vaziri F, Siadat SD. 2017. Evaluation of the impact of polyclonal infection and heteroresistance on treatment of tuberculosis patients. Sci Rep 7:1–4. doi:10.1038/srep41410. 28120910 PMC5264600

[4] Cohen T, Colijn C, Murray M. 2008. Modeling the effects of strain diversity and mechanisms of strain competition on the potential performance of new tuberculosis vaccines. Proc Natl Acad Sci USA 105:16302–16307. doi:10.1073/pnas.0808746105. 18849476 PMC2570977

[5] Wlodarska M, Johnston JC, Gardy JL, Tang P. 2015. A microbiological revolution meets an ancient disease: improving the management of tuberculosis with genomics. Clin Microbiol Rev 28:523–539. doi:10.1128/CMR.00124-14. 25810419 PMC4402953

[6] Baik Y, Modongo C, Moonan PK, Click ES, Tobias JL, Boyd R, Finlay A, Oeltmann JE, Shin SS, Zetola NM. 2020. Possible transmission mechanisms of mixed Mycobacterium tuberculosis Infection in high HIV prevalence country, Botswana. Emerg Infect Dis 26:953–960. doi:10.3201/eid2605.191638. 32310078 PMC7181944

[7] Mallard K, McNerney R, Crampin AC, Houben R, Ndlovu R, Munthali L, Warren RM, French N, Glynn JR. 2010. Molecular detection of mixed infections of Mycobacterium tuberculosis strains in sputum samples from patients in Karonga District, Malawi. J Clin Microbiol 48:4512–4518. doi:10.1128/JCM.01683-10. 20962138 PMC3008455

[8] Huyen MN, Kremer K, Lan NT, Cobelens FG, Buu TN, Dung NH, Caws M, Tiemersma EW, van Soolingen D. 2012. Mixed tuberculosis infections in rural South Vietnam. J Clin Microbiol 50:1586–1592. doi:10.1128/JCM.00434-12. 22378903 PMC3347091

[9] Shin SS, Modongo C, Baik Y, Allender C, Lemmer D, Colman RE, Engelthaler DM, Warren RM, Zetola NM. 2018. Mixed Mycobacterium tuberculosis-strain infections are associated with poor treatment outcomes among patients with newly diagnosed tuberculosis, independent of pretreatment heteroresistance. J Infect Dis 218:1974–1982. doi:10.1093/infdis/jiy480. 30085153 PMC6217728

[10] Byrne AS, Goudreau A, Bissonnette N, Shamputa IC, Tahlan K. 2020. Methods for detecting mycobacterial mixed strain infections: a systematic review. Front Genet 11:600692. doi:10.3389/fgene.2020.600692. 33408740 PMC7779811

[11] Meehan CJ, Goig GA, Kohl TA, Verboven L, Dippenaar A, Ezewudo M, Farhat MR, Guthrie JL, Laukens K, Miotto P, Ofori-Anyinam B, Dreyer V, Supply P, Suresh A, Utpatel C, van Soolingen D, Zhou Y, Ashton PM, Brites D, Cabibbe AM, de Jong BC, de Vos M, Menardo F, Gagneux S, Gao Q, Heupink TH, Liu Q, Loiseau C, Rigouts L, Rodwell TC, Tagliani E, Walker TM, Warren RM, Zhao Y, Zignol M, Schito M, Gardy J, Cirillo DM, Niemann S, Comas I, Van Rie A. 2019. Whole genome sequencing of Mycobacterium tuberculosis: current standards and open issues. Nat Rev Microbiol 17:533–545. doi:10.1038/s41579-019-0214-5. 31209399

[12] Gan M, Liu Q, Yang C, Gao Q, Luo T. 2016. Deep whole-genome sequencing to detect mixed infection of Mycobacterium tuberculosis. PLoS One 11:e0159029. doi:10.1371/journal.pone.0159029. 27391214 PMC4938208

[13] Anyansi C, Keo A, Walker BJ, Straub TJ, Manson AL, Earl AM, Abeel T. 2020. QuantTB: a method to classify mixed Mycobacterium tuberculosis infections within whole genome sequencing data. BMC Genomics 21:80. doi:10.1186/s12864-020-6486-3. 31992201 PMC6986090

[14] de Beer JL, Akkerman OW, Schürch AC, Mulder A, van der Werf TS, van der Zanden AG, van Ingen J, van Soolingen D. 2014. Optimization of standard in-house 24-locus variable-number tandem-repeat typing for Mycobacterium tuberculosis and its direct application to clinical material. J Clin Microbiol 52:1338–1342. doi:10.1128/JCM.03436-13. 24501023 PMC3993658

[15] Zentilin L, Giacca M. 2007. Competitive PCR for precise nucleic acid quantification. Nat Protoc 2:2092–2104. doi:10.1038/nprot.2007.299. 17853864

[16] Ford C, Yusim K, Ioerger T, Feng S, Chase M, Greene M, Korber B, Fortune S. 2012. Mycobacterium tuberculosis: heterogeneity revealed through whole genome sequencing. Tuberculosis (Edinb) 92:194–201. doi:10.1016/j.tube.2011.11.003. 22218163 PMC3323677

[17] Jajou R, de Neeling A, van Hunen R, de Vries G, Schimmel H, Mulder A, Anthony R, van der Hoek W, van Soolingen D. 2018. Epidemiological links between tuberculosis cases identified twice as efficiently by whole genome sequencing than conventional molecular typing: a population-based study. PLoS One 13:e0195413. doi:10.1371/journal.pone.0195413. 29617456 PMC5884559

[18] Meehan CJ, Moris P, Kohl TA, Pečerska J, Akter S, Merker M, Utpatel C, Beckert P, Gehre F, Lempens P, Stadler T, Kaswa MK, Kühnert D, Niemann S, de Jong BC. 2018. The relationship between transmission time and clustering methods in Mycobacterium tuberculosis epidemiology. EBioMedicine 37:410–416. doi:10.1016/j.ebiom.2018.10.013. 30341041 PMC6284411

[19] Sobkowiak B, Glynn JR, Houben RMGJ, Mallard K, Phelan JE, Guerra-Assunção JA, Banda L, Mzembe T, Viveiros M, McNerney R, Parkhill J, Crampin AC, Clark TG. 2018. Identifying mixed Mycobacterium tuberculosis infections from whole genome sequence data. BMC Genomics 19:613. doi:10.1186/s12864-018-4988-z. 30107785 PMC6092779

[20] Gabbassov E, Moreno-Molina M, Comas I, Libbrecht M, Chindelevitch L. 2021. SplitStrains, a tool to identify and separate mixed Mycobacterium tuberculosis infections from WGS data. Microb Genom 7:e000607. doi:10.1099/mgen.0.000607. PMC8461467 34165419

[21] Anyansi C, Straub TJ, Manson AL, Earl AM, Abeel T. 2020. Computational methods for strain-level microbial detection in colony and metagenome sequencing data. Front Microbiol 11:1925. doi:10.3389/fmicb.2020.01925. 33013732 PMC7507117

[22] Goossens SN, Heupink TH, De Vos E, Dippenaar A, De Vos M, Warren R, Van Rie A. 2022. Detection of minor variants in Mycobacterium tuberculosis whole genome sequencing data. Brief Bioinform 23:bbab541. doi:10.1093/bib/bbab541. 34962257 PMC8769888

[23] Nimmo C, Brien K, Millard J, Grant AD, Padayatchi N, Pym AS, O’Donnell M, Goldstein R, Breuer J, Balloux F. 2020. Dynamics of within-host Mycobacterium tuberculosis diversity and heteroresistance during treatment. EBioMedicine 55:102747. doi:10.1016/j.ebiom.2020.102747. 32361247 PMC7195533

[24] Fang R, Li X, Li J, Wu J, Shen X, Gui X, DeRiemer K, Liu L, Mei J, Gao Q. 2008. Mixed infections of Mycobacterium tuberculosis in tuberculosis patients in Shanghai, China. Tuberculosis (Edinb) 88:469–473. doi:10.1016/j.tube.2008.02.002. 18424179 PMC4391511

[25] Luo T, Yang C, Pang Y, Zhao Y, Mei J, Gao Q. 2014. Development of a hierarchical variable-number tandem repeat typing scheme for Mycobacterium tuberculosis in China. PLoS One 9:e89726. doi:10.1371/journal.pone.0089726. 24586989 PMC3934936

[26] Peng Y, Yang C, Li X, Luo T, Li F, Gao Q. 2013. Multiple samples improve the sensitivity for detection of mixed Mycobacterium infections. Tuberculosis (Edinb) 93:548–550. doi:10.1016/j.tube.2013.06.002. 23838118

[27] Chen Y, Ji L, Liu Q, Li J, Hong C, Jiang Q, Gan M, Takiff HE, Yu W, Tan W, Gao Q. 2021. Lesion heterogeneity and long-term heteroresistance in multidrug-resistant tuberculosis. J Infect Dis 224:889–893. doi:10.1093/infdis/jiab011. 34467983

[28] Cohen T, Chindelevitch L, Misra R, Kempner ME, Galea J, Moodley P, Wilson D. 2016. Within-host heterogeneity of Mycobacterium tuberculosis infection is associated with poor early treatment response: a prospective cohort study. J Infect Dis 213:1796–1799. doi:10.1093/infdis/jiw014. 26768249 PMC4857469

[29] Asare-Baah M, Seraphin MN, Salmon LAT, Lauzardo M. 2021. Effect of mixed strain infections on clinical and epidemiological features of tuberculosis in Florida. Infect Genet Evol 87:104659. doi:10.1016/j.meegid.2020.104659. 33276149 PMC7855629

[30] Kamakoli MK, Farmanfarmaei G, Masoumi M, Khanipour S, Gharibzadeh S, Sola C, Fateh A, Siadat SD, Refregier G, Vaziri F. 2020. Prediction of the hidden genotype of mixed infection strains in Iranian tuberculosis patients. Int J Infect Dis 95:22–27. doi:10.1016/j.ijid.2020.03.056. 32251801

[31] Séraphin MN, Norman A, Rasmussen EM, Gerace AM, Chiribau CB, Rowlinson MC, Lillebaek T, Lauzardo M. 2019. Direct transmission of within-host Mycobacterium tuberculosis diversity to secondary cases can lead to variable between-host heterogeneity without de novo mutation: a genomic investigation. EBioMedicine 47:293–300. doi:10.1016/j.ebiom.2019.08.010. 31420303 PMC6796532

[32] Larsen MH, Biermann K, Tandberg S, Hsu T, Jacobs WR Jr. 2007. Genetic manipulation of Mycobacterium tuberculosis. Curr Protoc Microbiol 6:10A.2. doi:10.1002/9780471729259.mc10a02s6. 18770603

[33] Yang T, Gan M, Liu Q, Liang W, Tang Q, Luo G, Zuo T, Guo Y, Hong C, Li Q, Tan W, Gao Q. 2022. SAM-TB: a whole genome sequencing data analysis website for detection of Mycobacterium tuberculosis drug resistance and transmission. Brief Bioinform 23:bbac030. doi:10.1093/bib/bbac030. 35211720 PMC8921607

[34] Kozlov AM, Darriba D, Flouri T, Morel B, Stamatakis A. 2019. RAxML-NG: a fast, scalable and user-friendly tool for maximum likelihood phylogenetic inference. Bioinformatics 35:4453–4455. doi:10.1093/bioinformatics/btz305. 31070718 PMC6821337

