# Supplementary material for: Whole-Genome Sequencing Exhibits Better Diagnostic Performance than Variable-Number Tandem Repeats for Identifying Mixed Infections of Mycobacterium tuberculosis
Source: Microbiol Spectr. 2023 Apr 26;11(3):e03570-22. doi: 10.1128/spectrum.03570-22 (PMC10269500; doi:10.1128/spectrum.03570-22)
Supplement: Supplemental file 1 — Supplemental material. Download spectrum.03570-22-s0001.pdf, PDF file, 1.0 MB [file spectrum.03570-22-s0001.pdf]

Supplementary materials

Table S1. Information on strains used in our laboratory mixed samples.

	**Group A**		**Group B**	
	Dominant strain (D)	Minor strain-1 (M1)	Dominant strain (D)	Minor strain-2 (M2)
Accession	ERR3559309	ERR3559282	ERR3559309	ERR3559369
Lineage	Lineage 2	Lineage 4	Lineage 2	Lineage 2
Pairwise distance	1159 bp		392 bp	

Table S2.Primer sequences used in this study for 9+3 VNTR loci.

**Locus**	**Primer pairs (5' to 3')**
**Conventional loci**	
QUB-11b	CGTAAGGGGGATGCGGGAAATAGG<br>CGAAGTGAATGGTGGCAT
QUB-18	ATCGTCAGCTGCGGAATAGT<br>AATACCGGGGATATCGGTTC
QUB-26	AACGCTCAGCTGTCGGAT<br>GGCCAGGTCCTTCCCGAT
MIRU-26	GCGGATAGGTCTACCGTCGAAATC<br>TCCGGGTCATACAGCATGATCA
MIRU-31	CGTCGAAGAGAGCCTCATCAATCAT<br>AACCTGCTGACCGATGGCAATATC
MIRU-40	GATTCCAACAAGACGCAGATCAAGA<br>TCAGGTCTTTCTCTCACGCTCTCG
Mtub-21	AGATCCCAGTTGTCGTCGTC<br>CAACATCGCCTGGTTCTGTA
Mtub-04	GTCCAGGTTGCAAGAGATGG<br>GGCATCCTCAACAACGGTAG

VNTR-2372	ACCTCCGTTCCGATAATC<br>CAGCTTTCAGCCTCCACA
**Hypervariable loci**	
VNTR-3820	TGCGCGGTGAATGAGACG<br>ACCTTCATCCTTGGCGAC
VNTR-4120	GTTCACCGGAGCCAACC<br>GAGGTGGTTTCGTGGTCG
VNTR-3232	CAGACCCGGCGTCATCAAC<br>CCAAGGGCGGCATTGTGTT

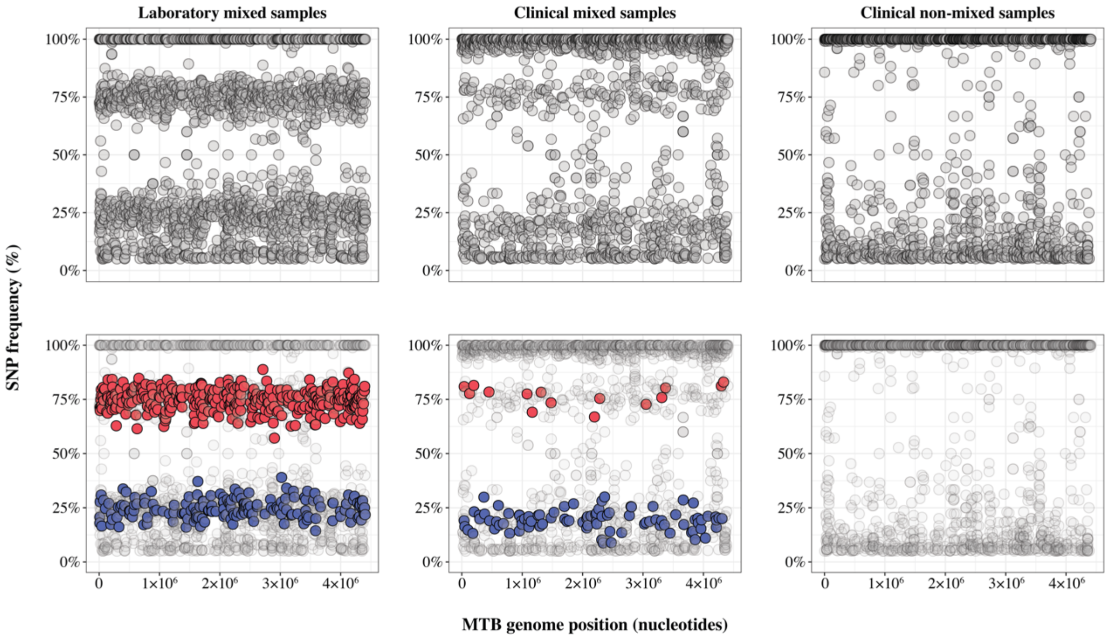

Figure S1. Principle of the phylogenomic-database based detection of mixed infection. The detection of mixed infection by phylogenetic-based method is depending upon the identification of divergence events (i.e., two strain specific paths) of two strains whose distinct evolutionary paths are both completely or partially included in the reference phylogenomic database. Therefore, if the divergence of two strains is not included in the database, the mixed infection will not be detected. Gray points indicate all SNPs, red points indicate the dominant strain specific SNPs determined by the reference phylogenomic database, and blue point indicates the minor strain specific SNPs determined by the reference phylogenomic database.

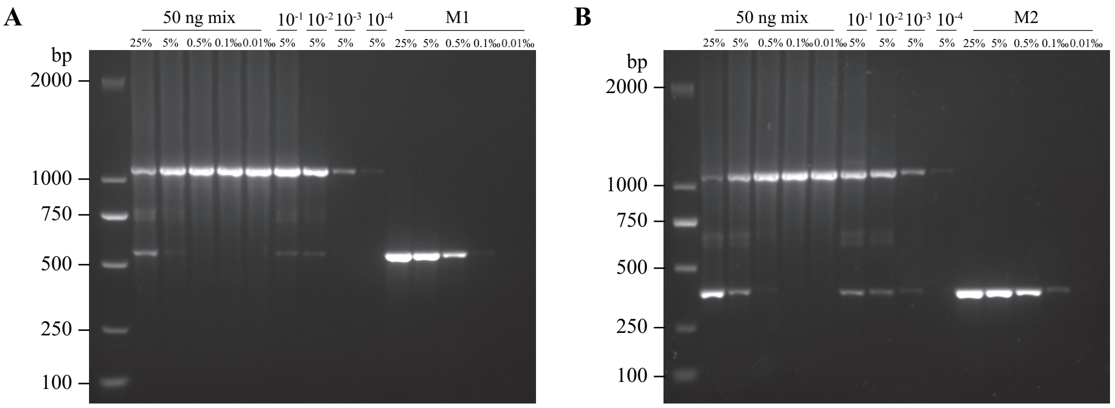

Figure S2. Examples of the resulting VNTR-PCR for laboratory samples. (A) PCR amplification of the VNTR loci (VNTR-3820) of artificial mixed samples consisting of two strains belonging to distinct phylogenetic lineages (Group A); PCR amplification of the VNTR loci (VNTR-3820) of 10-fold serial dilutions of artificial mixed samples (Group A); PCR amplification of the VNTR loci (VNTR-3820) of the minor strain alone, based on DNA quantities corresponding to the relevant mixed samples. (B) PCR amplification of the VNTR loci (VNTR-3820) of artificial mixed samples consisting of two strains belonging to the same lineage (Group B); PCR amplification of the VNTR loci (VNTR-3820) of 10-fold serial dilutions of artificial mixed samples (Group B); PCR amplification of the VNTR loci (VNTR-3820) of the minor strain alone, based on DNA quantities corresponding to the relevant mixed samples.
